# Osteoid Osteoma of Distal Phalanx of Toe: A Rare Cause of Foot Pain

**DOI:** 10.1155/2014/560892

**Published:** 2014-09-24

**Authors:** Hakan Başar, Osman Mert Topkar, Bülent Erol

**Affiliations:** ^1^Department of Orthopedics Surgery, Sakarya Training and Research Hospital, Eski Kazımpaşa Caddesi Yolu Arabacıalanı Mah. Akkent Villaları No. 156/25, Serdivan, 54050 Sakarya, Turkey; ^2^Department of Orthopaedic Surgery, Marmara University School of Medicine, Istanbul, Turkey

## Abstract

Osteoid osteoma is an uncommon benign tumor and causes severe pain, being worse at night, that responds dramatically to nonsteroidal anti-inflammatory medications. An osteoid osteoma of the toe is very rare and arising in a pedal phalanx may be difficult to diagnose. A 34-year-old male has local swelling and tenderness but there were no hyperemia, temperature increase, or clubbing. There was a 2-month history of antibiotic treatment with suspicion of soft tissue infection in another clinic. The osteoid osteoma was completely excised by curettage and nidus removal with open surgical technique. The patient was followed up for 63 months with annual clinical and radiographic evaluations. There was no relapse of the pain and no residual recurrent tumour. Osteoid osteoma may be difficult to distinguish from chronic infection or myxedema. The patients may be taken for unnecessary treatment. The aim of the treatment for osteoid osteoma is to remove entire nidus by open surgical excision or by percutaneous procedures such as percutaneous radiofrequency and laser ablation. Osteoid osteomas having radiologic and clinical features other than classical presentation of osteoid osteoma are called atypical osteoid osteomas. Atypical localized osteoid osteomas can be easily misdiagnosed and treatment is often complicated.

## 1. Introduction

Osteoid osteoma is an uncommon benign tumor of the skeleton that usually occurs in the second and third decades of life [1]. This benign tumor causes severe pain, worse at night, that responds dramatically to nonsteroidal anti-inflammatory medications [2]. An osteoid osteoma of the toe is very rare. Therefore, osteoid osteoma arising in a pedal phalanx may be difficult to diagnose [3]. Clinical and radiological differentiation from subacute osteomyelitis is difficult [4]. Swelling, erythema, and enlargement may be seen in the toe in additional severe pain. We report an uncommon case of osteoid osteoma located at the distal phalanx of the hallux.

## 2. Case Report

A 34-year-old male was admitted to our clinic with complaints of progressive and severe localized pain at the tip of the toe of the right foot. The patient had ongoing pain during 17 months, particularly at night. The pain was relieved by nonsteroidal anti-inflammatory drugs. The patient was not able to wear a shoe due to the pain. The toe of the patient demonstrated local swelling and tenderness but there was no hyperemia or temperature increase. There was also no history of trauma. The patient has received 2 months of antibiotic treatment at another hospital because of suspicion of soft tissue infection.

Our patient presented with characteristic symptoms of night pain that was temporarily relieved by nonsteroidal anti-inflammatory drug (NSAID), local swelling of the digits, and typical radiographic images of nidus formation. Osteoid osteomas usually cause clubbing deformity when diagnosed at the finger [5]. But our patient did not have a clubbed toe. The diagnosis of osteoid osteoma was made preoperatively according to the clinical and radiological evaluations.

**Figure 1:**
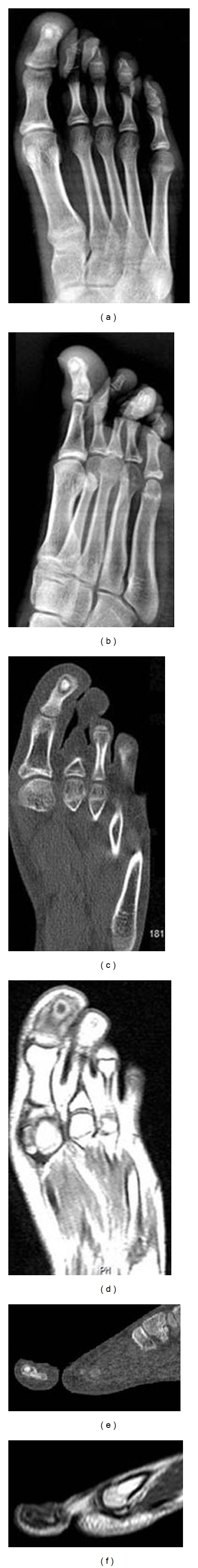
Preoperative X-rays. (a, b) Computerized tomography (CT) images (c, e) and Magnetic resonance imaging (MRI) (d, f) which revealed a small oval radiolucency with cortical sclerosis, nidus formation, and cortical defect at the tip of the distal phalanx.

X-rays, BT, and MR images revealed a small oval radiolucency with cortical sclerosis, nidus formation, and a cortical defect at the tip of the distal phalanx. Radiography was very important to make the diagnosis of an osteoid osteoma in this patient (Figure 1).

**Figure 2:**
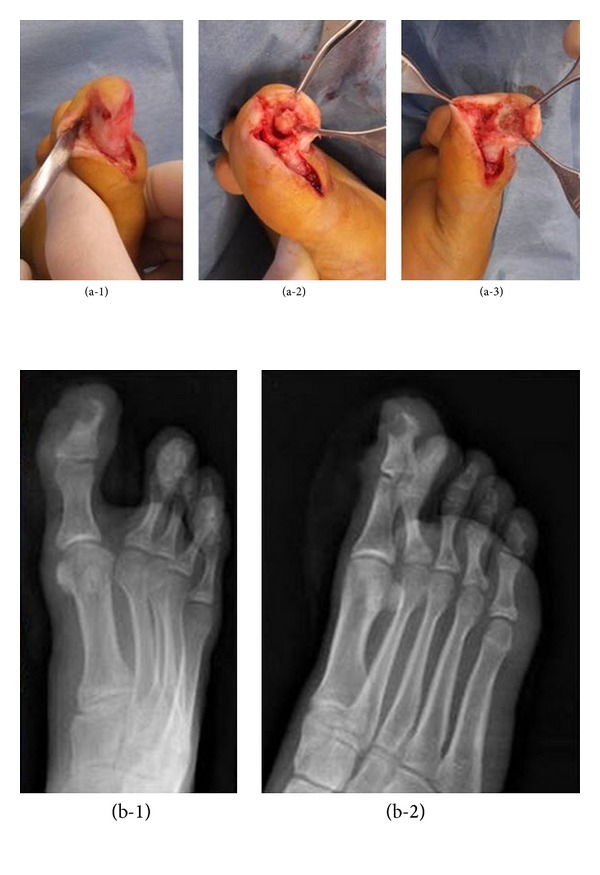
(a-1) Longitudinal incision at medial side of the distal phalanx of the toe. (a-2) Dissection of the osteoid osteoma. (a-3) Curettage of the osteoid osteoma. (b-1, b-2) Postoperative radiographies.

A longitudinal incision at the medial side of the distal phalanx of the toe was preferred for surgical exposure of the tumour. The mass was completely excised by curettage and nidus removal (Figure 2). Soft and bony tissue samples were obtained for histopathological examination and microbiological culture. Microbiological cultures revealed no pathogenic organism. The patient's pain resolved immediately after the operation. The diagnosis was pathologically confirmed. The patient was followed up for 63 months with annual clinical, radiographic evaluations. There were no signs of the residual/recurrent disease.

## 3. Discussion

Osteoid osteoma is a benign osteoblastic tumor [6]. It occurs most frequently in the lower extremity at the meta-diaphyseal region of the long bones in younger individuals. The incidence is 2–11% for the ankle and foot [7]. The talus is the most commonly affected bone in the foot [8]. An osteoid osteoma of the toe is very rare. Therefore, osteoid osteoma arising in a pedal phalanx may be difficult to diagnose [3]. Classically, osteoid osteoma presents with progressive night pain that is temporarily relieved by aspirin or NSAID [9]. Phalangeal lesions are typically small in size and cause local tenderness. Clubbing of the finger and toe with nail hypertrophy is presented when the lesion affects the distal phalanx [1]. Digital clubbing is a common finding when osteoid osteoma localizes to the distal phalanx of toe [1].

It may be difficult to distinguish osteoid osteoma from chronic infection or myxedema. The patients may be taken for unnecessary treatment. The clinical and radiological evaluations are very important in diagnosis for osteoid osteoma. Subacute infection, osteoblastoma, epidermoid inclusion cyst, subungual exostosis, glomus tumor, enchondroma, chondroblastoma, and chondromyxoid fibroma should be considered in the other differential diagnosis [6, 7, 9]. Histopathologic evaluation is very important in establishing the definitive diagnosis.

Our patient presented with characteristic symptoms of night pain that was temporarily relieved by NSAID, local swelling of the digit, and a radiographically typical nidus. But our patient did not have clubbed toe. The diagnosis of osteoid osteoma was made preoperatively by clinical and radiographic evaluation.

A nidus of an osteoid osteoma contains vascular osteoid, which may show mineralization. Surrounding reactive bony sclerosis can make the nidus obvious on radiography. Typically, radiography shows a small lytic lesion with surrounding sclerotic change and nidus formation [10]. Most osteoid osteomas are diagnosed based on progressive night pain and radiographic images [10].

Traditionally, the treatment for osteoid osteoma is to remove the entire nidus. Types of surgical resection of osteoid osteoma are open surgical excision and percutaneous procedures. Percutaneous procedures are surgical resection [11], ethanol injection [12], radiofrequency ablation [13], and laser photocoagulation [14]. Percutaneous treatments of osteoid osteomas of the foot have some complications as injury to neurovascular structures, skin and soft tissue damage, or osteonecrosis of the bones [15]. The main disadvantage of these percutaneous procedures such as percutaneous radiofrequency and laser ablation has the lack of histopathologic confirmation. For surgical complications and lack of histological confirmation, we preferred open surgical excision. We did not observe any postprocedural complications.
